# Recent Developments in Preclinical DNA Vaccination

**DOI:** 10.3390/vaccines2010089

**Published:** 2014-01-13

**Authors:** Kenji Okuda, Yoshiyuki Wada, Masaru Shimada

**Affiliations:** 1 Department of Molecular Biodefense Research, Yokohama City University Graduate School of Medicine, Yokohama, Kanagawa Prefecture 236-0004, Japan; E-Mail: kqxin@yahoo.co.jp; 2 Choju Medical Institute, Toyohashi, Aichi Prefecture 441-8124, Japan; 3 Vaccine Institute, Yokohama, Kanagawa Prefecture 2235-0045, Japan; E-Mail: yoshiyukiw@hotomail.com

**Keywords:** DNA vaccine, genetic adjuvants, electroporation

## Abstract

The advantages of genetic immunization of the new vaccine using plasmid DNAs are multifold. For example, it is easy to generate plasmid DNAs, increase their dose during the manufacturing process, and sterilize them. Furthermore, they can be stored for a long period of time upon stabilization, and their protein encoding sequences can be easily modified by employing various DNA-manipulation techniques. Although DNA vaccinations strongly increase Th1-mediated immune responses in animals, several problems persist. One is about their weak immunogenicity in humans. To overcome this problem, various genetic adjuvants, electroporation, and prime-boost methods have been developed preclinically, which are reviewed here.

## 1. Introduction

DNA vaccination represents a novel means of expressing *in vitro* antigens for the generation of both humoral and cellular immunities against a wide spectrum of infectious agents, including viruses, bacteria, parasites, and tumors [1,2,3,4]. These vaccines have elicited protective immunity in a number of preclinical disease models [5,6,7,8,9]. DNA vaccines contain genes encoding the protein of the pathogen itself. Such plasmids neither replicate in the mammalian host nor integrate themselves within the chromosomal DNA.

DNA vaccination was first demonstrated when young mice were inoculated with a plasmid expressing human growth hormone (hGH) [10]. In the initial experiment, the *hGH* gene was injected in the skin of the ear, with the intention of synthesizing the hGH protein for gene therapy. Several immunized mice showed substantial levels of antibodies, two months after the injection of the plasmid DNA (pDNA) into their cells, where the pDNA was read and the hGH protein was synthesized. Similar to the immune responses observed in a viral infection or an attenuated virus vaccination, the intracellular production of protein or peptide antigen induces high levels of Th1-type responses. However, due to the limited amount of protein synthesis in the body, the Th2-type immune responses are elicited at low levels. 

Although some experimental trials aimed at facilitating clinical or preclinical studies have evoked an immune response against microbial diseases, the usefulness of this technique has not been conclusively proved in multiple animal models [4]. A DNA vaccine to protect horses from West Nile virus, as well as other DNA Vaccines, has been approved for veterinary use [11,12,13]. Bar-Or and coworkers [14] successfully elicited antigen-specific tolerance, with a DNA vaccine encoding myelin basic protein, in patients with multiple sclerosis (MS). Although MS is known to be a demyelinating neurodegenerative disorder of the central nervous system in humans [14,15,16], the mechanism underlying the activity of this vaccine is yet to be fully resolved. 

DNA vaccines have many potential benefits despite their weak immune responses in humans. Methods such as *in vivo* electroporation have improved the efficacy of these vaccines. However, an optimal strategy for safe, reproducible, and pain-free DNA vaccination is yet to be developed.

## 2. Methods of Constructing DNA Vaccine

DNA vaccines elicit good levels of immune response when highly expressing vectors are used. These plasmids usually consist of a strong viral promoter to drive the *in vivo* transcription and translation of the gene. 

### 2.1. Promoters and Enhancers

Several promoters or enhancers are sometimes included to improve mRNA stability and increase protein expression. Plasmids also include a strong transcriptional termination signal [1,2,3]. Some multicistronic vectors are designed to express several immunogens or to express a number of immunostimulatory proteins.

An effective vector design is important for maximal protein expression [16]. Optimizing the codon usage of mRNAs in eukaryotic cells is an ideal way to enhance protein expression. As pathogens often show different AT codon usages, changes in the gene sequence to enhance the more effective codons in the target species improve the gene expression. 

Another important factor in the construction of DNA vaccines is the choice of the promoter. Although the SV40 promoter has been widely used [2], the expression rate of DNA vaccines has been increased using the cytomegalovirus (CMV) immediate early promoter [1,2]. Furthermore, several studies showed that a chimera CMV promoter with a chicken β-actin intron (CAG promoter) generates a higher expression of CMV protein by several folds [2,4,17]. 

Inclusion of the *cis*-acting transcriptional elements (CTE) from monkey virus with rev increased the envelope expression. The *rev*+CTE constructs have distinct advantages for increasing the expression rates. When an envelope sequence from East-African subtype A virus was inserted into an expression vector, it increased the expression rates of the CMV promoter [1,2,17]. Additional modifications to increase the expression rates include the insertion of enhancer sequences, synthetic introns, and adenovirus tripartite leader sequences, and modifications to polyadenylation and transcriptional termination sequences. 

### 2.2. Insertion of DNA Sequences

The design of the DNA sequences or the optimized codon usages can be targeted to various cellular compartments to improve the Th1 or Th2 responses. The addition of *N*-terminal ubiquitin signals increases immunogenicity [4,11,18], while a conformational change of the amino acids in the protein sometimes enhances the immune responses. The optimized codon usage also enhances protein expression [1,2,18].

## 3. Delivery Method of Plasmid (p)DNA

DNA vaccines have been used to immunize animals by using a number of methods. The type of T-cells raised is influenced by the method of delivery, type of immunogen expressed, as well as targeting of different lymphoid cells. Each method has distinct advantages for immune activation. However, no single method was successful in enhancing the protective effect and properly regulating the induction of a steady immune response.

### 3.1. Immunization by Needles

Generally, needle injections are used to induce Th1 responses. Ulmer *et al.* [19] demonstrated that pDNA encoding viral nucleoprotein (NP) of influenza virus could induce cytotoxic activity regardless of the viral antigen shifts and consequently protect animals from lethal infection. Numerous studies have revealed that these needle injections of pDNA are very effective against viral infections in animal models [2,4,20,21]. The popular approaches for the injection of pDNA by using a needle involve the use of bupivacaine, hypotonic solution, or saline, or sometimes the use of an electroporation method. Immunization is usually performed by intramuscular injection in the skeletal muscles, or by intradermal injection, with the DNA being delivered to the extracellular spaces or into the cells. The delivery into the cells is assisted in some cases by electroporation [22], or by temporarily damaging muscular fibers with bupivacaine or hypertonic solutions. Hence, it is rather difficult to obtain a constant immune response [2]. In addition, the immune responses to these needle methods are also affected by many factors, including needle type, muscle type, age of the animal, and the speed of injection [2,3]. 

The rapid implantation of vaccine-loaded polymer films is used for carrying DNA and biodegradable polycations into the epidermis, which is rich in immune cells, using microneedles coated with releasable polyelectrolyte multilayers. Films transferred into the skin following a brief microneedle application promoted local transfection and controlled the persistence of DNA and adjuvants in the skin for weeks. These “multilayer tattoo” DNA vaccines induced immune responses against a model HIV antigen, which were comparable to electroporation in mice. These vaccines also elicited an increase in the gene expression in non-human primate skin, which was 140-fold higher than the level elicited by an intradermal DNA injection [22,23]. At present, this type of needle injection is widely used in animal studies.

### 3.2. The Gene Gun Method

The gene gun is often used as it increases the Th2 responses [24,25,26]. The pDNA that has been coated on the surface of the gold, is introduced into the cells by using compressed helium as an accelerant [2,24]. This method is effective as it takes advantage of the molecular weight and the safety of gold. Saline injections require 2–20 μg pDNA per mice, whereas gene gun deliveries require only 1–3 μg pDNA to increase an effective immune response [24,25,26]. Most of these results were observed using mice. For clinical trials in humans, it is important to decrease the dose of pDNA because the quantities vary from species to species. For example, primates require approximately 10 times more pDNA than mice. Moreover, saline injections require more pDNA because the pDNA is delivered to extracellular spaces of the target tissue (normally, muscle cells), whereas gene gun injects pDNA directly into the cells [26]. Due to the weak immunogenicity of pDNA, this immunization method must be performed several times and in many places to elicit a potent immune response. 

### 3.3. Intranasal (i.n.) Administrations

Another delivery method is the i.n. inhalation of pDNA through the nasal and lung mucosa. Tadokoro *et al.* [27] investigated tissue distribution of DNA plasmids by i.n. administration, using a fluorescence *in situ* hybridization (FISH) method. Their study revealed that the DNA plasmids localized in the alveoli of the lung, liver, spleen, regional lymph nodes, kidney, fetus, and esophagus in the administrated mice. The HIV plasmids were detected two to four weeks after administration. The messenger RNA of HIV env gene was detected in the lung, liver, and spleen, while type 1 HIV proteins were detected in the lungs [27]. DNA vaccination by i.n. as well as intravaginal administration of constructs with HIV genes induced high levels of Th2 immune responses against HIV antigens. The level of mucosal IgA antibodies detected in the feces and vaginal fluid was significant in i.n. administration. This route of administration also resulted in significant levels of HIV-1-neutralizing antibodies in feces and serum [28]. In addition, i.n. immunization with the hemagglutinin gene of influenza pDNA induced a protective immune response against influenza virus in mice models. In addition, secretary IgA antibody was produced at significant levels by high doses of i.n. DNA vaccination in mice [29]. Cytokine assays revealed that i.n. administration of this DNA vaccine induced mainly Th2 immune responses [2,12,28]. A major advantage of the i.n. administration is the ability to increase the dose in order to enable several applications for a more effective DNA vaccination in mice [29,30]. One important point of the i.n. method is the strong induction of secretory IgA antibody. 

### 3.4. Topical Application and Other Routes of Immunization

The topical application of DNA vaccine to the skin is a useful method of immunization because of its simplicity, painlessness, and cost-effectiveness. However, the levels of immune responses it elicits are relatively low. Liu *et al.* [31] administered HIV-1 DNA vaccine with cytokine-expressing plasmids to the skin of mice by a new topical application technique involving prior elimination of keratinocytes by using fast-acting adhesives. Their findings revealed that the topical application of HIV-1 DNA vaccine induced an immune response against HIV-1 envelope antigen. Skin biopsy of the immunized mice showed significant activation of dendritic cells (DCs), suggesting that the topical application method is an efficient route of DNA vaccination [31]. 

Watabe *et al.* [32] reported the expression of a matrix (M) gene of the influenza virus by applying the DNA vaccine several times on the mouse skin, after removal of the keratinocytic layers. Immunization using this method induced M-specific antibodies and cytotoxic T lymphocyte (CTL) response to acquire resistance against the influenza virus. They further found that simultaneous topical application of GM-CSF expression plasmids or liposomes plus mannan produced a stronger immune response and enhanced the protective effect [28]. The ocular inflammatory disease has been treated by topical administration of pDNA encoding IL-10 or other genetic adjuvants [33]. 

DNA vaccination against herpes simplex virus-2 (HSV-2) by administration through vaginal mucosa has also been reported [34]. In addition, this study was the report of the DNA vaccination through the vaginal region by using Nanopatch^TM^ in mice systems [34]. These studies suggested that these immunizations are useful for mice against HSV-2 infection or might be effective in HIV infection. Mucosal surface delivery has also been achieved using cationic liposome-DNA preparations [3] and biodegradable microspheres [3,16]. 

We previously reported that in mice, the immune responses were transferred into the offspring when the mothers were intravenously injected with liposome-encapsulated DNA vaccine [35]. Though the uptake of pDNA was observed in the fetuses, the actual transfer occurred only during early pregnancy. During the early days after conception with cationic liposomes, the injected plasmid was detected in the tissues of the fetus, consistent with a transplacental transfer. Offspring mounted stronger antigen-specific immune responses than controls and they were protected against homologous influenza virus after vaccination. Moreover, such immune responses were stronger in the offspring when the mothers were injected with DNA plasmid during the early days after coitus. These results suggest that DNA-vaccinated mothers confer the antigen-specific immunity to their progeny.

### 3.5. CO_2_-Powered Biojector

The delivery of DNA vaccines by the needle-free Biojector® device induces Th2-type immune response as well as IFN-gamma ELISPOT and CD8^+^ T cell responses, when boosted with recombinant adenovirus or vaccinia virus vector vaccine. The needle-free delivery of DNA using a CO_2_-powered Biojector^®^ device was found to be a useful method of immunization.

Graham *et al.* [36] reported that the immune response to this CO_2_-powered Biojector^®^ method is enhanced by boosting with recombinant Ad5 vaccines. Although the side effects are minimal, this method is stronger than needle injection in human phase I trial. In a small number of human trials, no HIV-specific antibody responses were detected, even as low-magnitude HIV-specific T-cell responses were detected in approximately 50% of the vaccinees. This initial product led to the development of a 4-plasmid multiclade HIV DNA vaccine, indicating that more effective techniques are necessary for the use of this needleless method [37].

### 3.6. Electroporation (EP) Method

The EP method induces one of the strongest Th1 responses. This method induces an immune response that is 10 times or more stronger than the response induced by other pDNA vaccination methods used to immunize animals [38]. However, this method has a drawback owing to the high voltage of electricity used, which delayed its application in clinical medicine [2,38,39]. Although some modified methods have been reported recently, more effective methods are needed.

The immunogenicity of DNA vaccine was increased by EP method in mice [40,41]. Zhou and coworkers [42] reported the binding of programmed death-1 (PD1) to its ligands expressed on dendritic cells (DCs), by fusing soluble PD1 with simian immunodeficiency virus (SIV) antigen. Intramuscular immunization (i.m.) via EP of the fusion DNA in mice resulted in consistently high frequencies of HIV-specific, multifunctional, long-lived cytotoxic CD8^+^ T cells and robust anti-SIV antibody titers. Soluble PD1-based vaccination potentiated CD8^+^ T cell responses by enhancing the antigen binding and uptake in DCs and activation in the draining lymph nodes. Their findings suggest that PD1-based DNA vaccination by EP method could be used against HIV and other pathogens.

Donate *et al.* [43] recently developed a non-invasive electrode known as the multi-electrode array (MEA), which lies flat on the surface of the skin without penetrating the tissue. They evaluated the MEA for its use in DNA vaccination by using hepatitis B virus infection model. The plasmid encoding hepatitis B surface antigen (HBsAg) was delivered intradermally with the MEA to the guinea pig skin. The results indicate an increase in protein expression following plasmid delivery using the MEA compared to injection alone. 

In another study, pDNA vaccination using skin EP elicited robust humoral and CD8^+^ T-cell immune responses while limiting the invasiveness of the delivery method. The authors of that study compared the ability of homologous prime/boost DNA vaccinations by skin EP and i.m. injection, to elicit immune responses by ELISPOT assay, and studied the complexity of CD4^+^ T-cell responses. They found that DNA vaccinations by skin EP and i.m. injection were capable of eliciting both single and multifunctional vaccine-specific CD4^+^ T cells, which is important for protection from HIV infection. Although the amount of DNA delivered by skin EP was five-fold lower, it elicited a significant increase in the magnitude of multiple-cytokine producers suggesting that the skin EP is a useful method of vaccination against various infectious agents [38,44].

Electroporation gene therapy has been used in preclinical and clinical trials of melanoma. Delivery of IL-12 by EP method resulted in significant necrosis of melanoma cells in a majority of treated tumors, and significant lymphocytic infiltration, as observed in the biopsies obtained from patients in several cohorts. In addition, the responses to untreated lesions suggested the induction of a systemic response following therapy [45]. Bordbar *et al.* [46] described the use of EP-mediated DNA immunization to identify important protective epitopes of the large VAR2CSA protein from *Plasmodium falciparum*, which has been implicated in the pathology of placental malaria. Immunization of mice and rabbit with DNA plasmids induced a high titer of antisera and also induced the generation of protective antibodies. The EP-mediated HIV DNA vaccine increased the HIV-specific cell-mediated immunity by a magnitude of 70-fold over that of HIV-specific cell-mediated immunity elicited by intramuscular injection, as measured by gamma interferon ELISPOT assay. Intracellular cytokine staining analysis for ELISPOT responders revealed both CD4^+^ and CD8^+^ T cell responses, with co-secretion of multiple cytokines. Delivery of a pDNA encoding IL-12 or IL-2 by using electroporation was demonstrated to be effective. 

This may be the first report of phase 1 clinical trial using the EP method [45]. 

## 4. Enhancing the Methods of DNA Vaccination

### 4.1. Cytokine Adjuvants

DNA immunization is able to raise a range of TH responses by inducing synthesis of a variety of cytokines. A major advantage of DNA vaccines is the ease with which they can be manipulated to modify the type of T-cell that influences a Th1 or Th2 response by addition of several cytokine plasmids.To develop a more potent DNA vaccine, immunomodulatory effects of the administration of IL-2, GM-CSF, IL-12, IFN-gamma, or various expression plasmids were investigated [47,48]. When the vaccine and expression plasmids were incorporated into cationic liposomes [47,49] and administered to mice, the antigen-specific delayed-type hypersensitivity response [48] and cytotoxic T lymphocyte activity were significantly increased. The expression of the cytokines increased for a week or more, when the vaccines were administered in a plasmid form. An analysis of serum HIV-1-specific IgG subclasses showed a significant drop in the IgG1/IgG2a ratio in the group that received the plasmid cytokine-vaccine combination. These results demonstrate that the IL-2 expression plasmid strongly enhances the HIV-1-specific immune response via activation of T helper type-1 cells [49]. Cytokine assays revealed that the HIV-1 DNA vaccine plus IL-12 plasmid induced mainly Th2 immune responses [29,49,50]. Co-administration of pro-inflammatory agents (various interleukins, TNF, and GM-CSF) and Th2-inducing cytokines enhance antibody responses [28], whereas pro-inflammatory agents and Th1-inducing cytokines decrease humoral responses and increase cytotoxic responses, which is more important in the protection of viral infections. Co-stimulatory molecules such as B7-1, B7-2, and CD40L are also sometimes administered. Stimulated macrophages secrete IL-12, IL-18, TNF-α, IFN-α, IFN-β, and IFN-γ, while stimulated B-cells secrete IL-6 and IL-12 [28,50,51].

Co-administration of cationic liposomes greatly enhanced the immune responses, and the antibodies against HIV-1 persisted for a long time. Co-administration of the DNA vaccine with IL-12- and GM-CSF-expressing plasmids induced high levels of HIV-specific CTLs and an increase in delayed-type hypersensitivity when administered even by the i.n. route using mice [28,29].

The advantages of using genetic adjuvants are their low cost, simplicity of administration, as well as the avoidance of unstable recombinant cytokines and potentially toxic “conventional” adjuvants (such as alum, calcium phosphate, monophosphoryl lipid A, cholera toxin, cationic and mannan-coated liposomes, QS21 [52], carboxymethylcellulose, and ubenimex). Plasmid encoding B7-1 (a ligand on APCs) has successfully enhanced the immune response in anti-tumor models. A mixture of plasmids encoding GM-CSF and the circumsporozoite protein has enhanced protection against subsequent challenges. GM-CSF was proposed to cause DCs to present antigen more efficiently and enhance IL-2 production and TH cell activation, thus driving the increased immune response [53]. The timing/frequency of administration, the dose, as well as the combination of genetic adjuvants are important factors. In addition, the optical gene expression and the amount of pDNA vaccination should also be considered. 

### 4.2. Chemical Adjuvants

Sasaki *et al.* [52] compared a DNA vaccine encoding env of HIV-1 and evaluated the QS-21 saponin adjuvant. Vaccination via the i.n. and i.m. routes elicited comparable systemic immune responses, and QS-21 consistently enhanced antigen-specific serum immunoglobulin G2a (IgG2a) production, delayed-type hypersensitivity reaction, and cytolytic activity of splenocytes. Secretory IgA production and cytolytic activity of the mesenteric lymph node cells were preferentially elicited by i.n. immunization, and QS-21 augmented these activities. The enhancement of humoral and cellular immune responses, by QS-21, was abrogated by treatment with anti-IL-2 and anti-IFN-gamma monoclonal antibodies.

Arai *et al.* [54] examined the adjuvant effect of 8-bromocyclic AMP (8 Br-cAMP) on an HIV-1 DNA vaccine. Administration of the DNA vaccine and 8 Br-cAMP combination, via i.m. and i.n. routes in mice, enhanced both HIV-1-specific humoral and cellular immunities when compared to immunization with the DNA vaccine alone. Furthermore, when administered via the i.n. route, the combination was found to strongly induce the production of secretory IgA antibody. The adjuvant effect of 8 Br-cAMP on the DNA vaccine probably occurs via enhancement of CMV promoter activity of the vaccine.

The efficiency of DNA immunization can be improved by stabilizing the DNA against degradation and by increasing the efficiency of the delivery of DNA into the antigen-presenting cells [3]. This has been demonstrated by coating biodegradable cationic microparticles with DNA. Such DNA-coated microparticles can be as effective at raising CTL as the recombinant vaccinia viruses, especially when mixed with alum. Particles that are 300 nm in diameter appear to be the most efficient for uptake by antigen-presenting cells [3].

### 4.3. Immunostimulatory CpG Motifs

Unmethylated CpG motifs are prevalent in bacterial but not vertebrate genomic DNAs. Mycobacterium DNA has been reported to increase the adjuvant reactions in cancers. Oligodeoxynucleotides containing CpG motifs activate host defense mechanisms, inducing active innate and acquired immune responses [55,56]. Bacterial stimulatory CpG (CpG-S) motifs frequently increase the immune responses. Additionally, CpG-S motifs are hypomethylated and this enhancement occurs only by using bacterial DNA. In contrast, nucleotide sequences that inhibit the activation of an immune response (termed CpG neutralizing or CpG-N) are frequently observed in eukaryotic genomes [55,56]. The innate system works synergistically with the adaptive immune system induced by DNA vaccination. CpG-S motifs induce polyclonal B-cell activation and enhance cytokine expression and secretion. 

The Toll-like receptor (TLR) family functions as a mediator of innate immunity. The human TLR9 (TLR9) expression in human immune cells correlates with the responsiveness to bacterial CpG motifs. Immunostimulatory CpG motifs induce the expression of the TLR9 protein in human non-responder cells of CpG motifs [57,58,59]. However, most of the currently reported evidences for the existence of immunostimulatory CpG motifs come from murine studies. Hence, experimental data from other species could provide vital clues, because different species may require different DNA-flanking sequences. 

### 4.4. Alphavirus Vectors

Recombinant alphavirus-based vectors have also been used to improve DNA vaccination efficiency. Against the Foot-and-mouth disease the model animal were reported to be effective in guinea pigs [60]. In addition, the alphavirus-based vector vaccine delivered by adenovirus has reported to induce sterile immunity against classical swine fever using rabbits and pigs [61]. The genes encoding the antigen of interest are inserted into the alphavirus replicon, replacing structural genes but leaving non-structural replicase genes intact. The Sindbis virus and Semliki Forest virus have been used to build recombinant alpha virus replicons. Unlike conventional DNA vaccinations, the alpha virus vectors kill transfected cells and are transiently expressed. This may be due to the high levels of protein expressed by this vector, replicon-induced cytokine responses, or replicon-induced apoptosis, leading to enhanced antigen uptake by dendritic cells.

Abdulhaqq *et al.* [62] studied novel therapeutic and prophylactic DNA vaccines. To improve their vaccine constructs, they employed methods of RNA/codon optimization and antigen consensus to enhance the expression and cellular/humoral cross-reactivity, respectively. In addition, they studied the potential of various molecular adjuvants to skew Th1/Th2 responses, enhance cellular/humoral responses, and improve protection in various animal models. Subsequently, they observed enhancement of immune responses by the electroporation method and by the use of genetic adjuvants.

GenScript is a venture company that designs and produces optimized genes that can alter both naturally occurring and recombinant gene sequences to achieve the highest possible levels of productivity in any given expression system. The OptimumGene™ algorithm manufactured by this company takes into consideration a variety of critical factors involved in different stages of protein expression, such as codon adaptability, mRNA structure, and various *cis* elements. 

### 4.5. RNA Vaccines

Highly pathogenic avian influenza viruses are a continuous threat to chicken and human beings. The recombinant vesicular stomatitis virus (VSV) vectors expressing HA of subtype H5 were generated to combat this threat. To comply with biosafety issues, the G gene was deleted from the VSV genome. The resulting vaccine vector VSVΔG (HA) was propagated on helper cells, resulting in the generation of trans-VSV G protein, and the chickens were vaccinated with a single intramuscular dose of the infectious replicon particles. Subsequent application of the same vaccine strongly boosted the humoral immune response and completely prevented the shedding of the target virus and transmission to sentinel birds. Subsequently, a self-amplifying RNA vaccine was developed [63,64,65].

The non-viral delivery of a 9 kb self-amplifying RNA encapsulated within lipid nanoparticles substantially increased immunogenicity, as compared with the delivery of unformulated RNA. This unique vaccine technology was found to elicit broad, potent, and protective immune responses that were comparable to those elicited by a viral delivery technology, but without the inherent limitations of viral vectors. Given the many positive attributes of nucleic acid vaccines, their results suggest that a comprehensive evaluation of non-viral technologies to deliver self-amplifying RNA vaccines is warranted [66].

### 4.6. Prime-Boost with Other Vaccines

Prime-boost strategies have been successful in inducing protection against malaria and simian immunodeficiency virus (SIV), as observed in many studies [67,68,69,70]. For boosting the recombinant protein, recombinant poxviruses, adenovirus type 5 [71], adenovirus type 5/35 [18,72], or other vaccines have been used [73,74]. Prime-boost strategies with recombinant protein have increased both the neutralizing antibody titer and the antibody avidity, and increased the persistence of weak HIV-1 envelope or other protein immunogens [3,75]. Recombinant virus boosts have been shown to be very effective for activating Th-1 responses [76]. Priming several times with DNA vaccine induces a weak but long-lasting immunogen in the host, while boosting with the recombinant virus or protein, induces a high level of immune responses.

Priming of mice with pDNA encoding *Plasmodium yoelii* circumsporozoite surface protein, followed by boosting with a recombinant vaccinia virus expressing the same protein, induced significantly higher levels of antibody and CTL activity and provided higher levels of protection, as compared to the observations in mice immunized and boosted with pDNA alone [68,69,75]. These responses were further enhanced with a mixture of cytokine plasmids and boosting with recombinant vaccinia virus. An effective prime-boost strategy for the macaca malarial parasite *P. knowlesi* has also been demonstrated. Rhesus monkeys were primed with a multicomponent, multistage malarial DNA vaccine [76]. The DNA vaccine encoded 2 liver-stage antigens: the circumsporozoite surface protein (PkCSP) and sporozoite surface protein 2 (PkSSP2). They were then boosted with a recombinant canarypox virus encoding all four antigens (ALVAC-4). Immunized monkeys developed antibodies against sporozoites and infected erythrocytes. Partial protection against sporozoite challenge was achieved, and the parasitemia was significantly reduced, compared to the observation in control monkeys. Although these models are not ideal for extrapolation to malarial treatment in humans, they are important for pre-clinical trials [77,78].

After DNA vaccinations, the boosting vaccines are usually a protein vaccine, a live-attenuated virus or a viral vector to help suppress or clear infections. The genetic optimization of synthetic plasmid constructs and their encoded antigens, *in vivo* electroporation-mediated vaccine delivery, as well as co-delivery with molecular adjuvants have collectively enhanced both transgene expression and the elicitation of vaccine-induced immunity. In addition, the development of prime-boost regimens has significantly contributed to DNA vaccine immunogenicity, and clinical trials using prime-boost vaccination are now in progress [79].

## 5. Discussion

Genetic immunization using pDNA has been studied for over 23 years with significant progress. Efficacy studies against many microbial infections by using different animal models have been reported. As the DNA vaccines are easy to prepare and quick to design, which facilitates their mass production, they are considered to be one of the most ideal vaccines. However, the efficacy and safety of these vaccines have not been comprehensively analyzed. Although the first DNA vaccine has been reported, the finer details regarding the method of vaccination, the adjuvant, and the genetic structure of the vaccine are still inconclusive. At present, one of the best combinations for DNA vaccination would be the use of electroporation with proper genetic adjuvant followed by boosting with attenuated virus vector vaccines. However, more clinical trials are needed to prove that DNA vaccination can induce the satisfactory level of effective immune responses in humans. 

The immune responses measured thus far were not as robust as anticipated from the preclinical studies. This may be due to the differences in the selection of infectious agents, as the antimicrobial immune responses are different for various infectious agents. For example, HIV-infected patients with high viral counts mounted a modest T-cell response with a DNA vaccine encoding several HIV antigens, resulting in no effect on viral count.

At present, DNA vaccines induce strong Th1 immune responses but weak Th2 responses. A combination of booster vaccines could be used to address this concern. The developments of more effective and safety clinical studies of delivery methods are expected. The Table 1 summarizes the advantage and disadvantage of presently reported methods of DNA vaccine. The protective immune responses in malarial infection are different as the protective epitopes are different at different stages of the protozoan parasite, which renders preparation of a proper vaccine difficult. In HIV and influenza infections, the surface antigenic epitopes change frequently, and the use of internal protein antigens might be useful in DNA vaccine development. In these cases, the protein sequences of internal and surface conservative regions should be used for the preparation of DNA vaccines. Hence, the major challenge is to develop DNA vaccines that are potent enough to comprehensively protect against these infections. To overcome these hurdles, more effective adjuvants, administration methods, or other boosting vaccines are needed.

**Table 1:** Advantages and disadvantages of DNA vaccine administration methods.

Delivery method	Advantage	Disadvantage
Needle injection (im)	Activate Th1 immune response	Muscle pain
	pDNA spreads widely	
	Most commonly studied	
	Large amount of DNA can be injected	
Gene Gun (id)	Small amount of DNA injected	Weak Th1 type immune response
	Dominance of Th2 type immune response	
Biojector injection (id)	Induces Th1 immune response	Weak Th2 type induction response
	Easy to administer	
Intranasal immunization	Easy to administer	Weak overall immunogenicity
	Secretory IgA production	
	Effective for lung immunity	
Electroporation (id, im)	High level of immune response	Possible risk due to high voltage
	Long duration of immune response	
DNA with adjuvants	Tilt to desired Th1 or Th2	Unknown side-effects
Prime-boost (id,im)	High levels of immune responses	Complicated for production and administration

## 6. Conclusions

The DNA vaccination is a new method and has various advantages and brings a new impact into the vaccine field. In this manuscript we mainly discussed the vaccine’s concept, history, method, and the preclinical issues of the DNA vaccine. In addition, many clinical trials are new being carried out and a more conclusive DNA vaccination for humans will hopefully appear soon.
